# Longitudinal follow-up study on fear of falling during and after rehabilitation in skilled nursing facilities

**DOI:** 10.1186/s12877-015-0158-1

**Published:** 2015-12-04

**Authors:** Jan H. M. Visschedijk, Monique A. A. Caljouw, Eduard Bakkers, Romke van Balen, Wilco P. Achterberg

**Affiliations:** Department of Public Health and Primary Care, Leiden University Medical Centre, PO Box 9600, 2300 RC Leiden, The Netherlands; Zorggroep Laurens, Rotterdam, The Netherlands; Zorginstellingen Pieter van Foreest, Delft, The Netherlands

**Keywords:** Fear of falling, Rehabilitation, Skilled nursing facility, Discharge, Instrumental activities of daily living

## Abstract

**Background:**

Fear of falling (FoF) is regarded as a major constraint for successful rehabilitation in older people. However, few studies have investigated FoF in vulnerable older people who rehabilitate in a skilled nursing facility (SNF). Therefore, this study measures the prevalence of FoF during and after rehabilitation and assesses differences between those with and without FoF. The relation between FoF and instrumental activities of daily living (IADL) after discharge was also assessed.

**Methods:**

In this longitudinal follow-up study, patients who rehabilitated in a SNF were assessed at admission and at 4 weeks after discharge. A one-item instrument was used to measure FoF at admission; based on their answer, the patients were divided into groups with no FoF and with FoF. To study FoF after discharge, the one-item instrument and the short Falls Efficacy Scale-International (FES-I) were used. IADL after discharge was assessed with the Frenchay Activities Index (FAI).

**Results:**

Of all participants, 62.5 % had FoF at admission. The participants with FoF were older, more often female, and had a higher average number of falls per week, more depressive symptoms and a lower level of self-efficacy. Four weeks after discharge, 82.1 % of the participants had FoF. IADL after discharge was considerably lower in patients with FoF (FAI of 27.3 vs. 34.8; *p* = 0.001).

**Conclusions:**

FoF is common among older persons who rehabilitate in SNF. FoF seems to be persistent and may even increase after rehabilitation, thereby hampering IADL after discharge. Interventions are needed to reduce FoF to ensure better outcomes in older patients rehabilitating in a SNF.

## Background

Fear of falling (FoF) among older persons can result in increased disability, restriction of activity and loss of functional independence [1, 2]. FoF is widespread among community-dwelling older persons and its prevalence is reported to range from 21 to 85 % [3, 4]. Among older people in long-term care, more than 50 % have FoF [1]. FoF is also common among older people who rehabilitate after a stroke, a hip fracture or other disease and is a major constraint for successful rehabilitation, predicting rehabilitation outcome at both discharge and follow-up [5, 6]. For patients with hip fracture, FoF may have an even greater impact on functional recovery than pain or depression [7].

FoF was first used in the context of the post-fall syndrome [8] and efforts have been made to operationalise this concept. Tinetti et al. describe FoF as “*a lasting concern about falling that leads to an individual avoiding activities that he/she remains capable of performing*” and operationalised FoF as a loss of self-efficacy to perform certain activities without falling [9]. Others relate FoF to deteriorated postural control [10]. FoF has been described more generally as a broader concept of intrinsic fear or worry about falling [11]. Although falls-related self-efficacy may involve a slightly different concept [12], the term is often used as a proxy for FoF. Falls efficacy scales assess ‘concern’ about falling, a term closely related to FoF but probably less intense and emotional [13]. Therefore, when operationalising FoF different instruments have been used to measure the psychological outcomes of falling [14].

In the Netherlands, after a short period of hospitalisation, many older persons with an acute decrease in function rehabilitate in a skilled nursing facility (SNF). Four main patient groups can be distinguished based on the underlying condition which requires rehabilitation, i.e. stroke, trauma, elective orthopaedic surgery (e.g. total hip or knee replacement), and ‘other’ (such as cardiac, respiratory and oncologic diseases). Unfortunately, FoF has rarely been studied in these groups of patients, even though most are vulnerable and have a high level of comorbidity and disability [15]. Moreover, as a result of a trauma or another serious event (e.g. a stroke or surgical procedure), these patients may be more susceptible to have FoF. This may hamper them in performing more complex activities after discharge, such as housekeeping, leisure activities and social interaction. Also, the relation between FoF and these so-called instrumental activities of daily living (IADL) has not been studied in these older patients.

Therefore, the present study aimed to assess FoF in different patient groups rehabilitating in a SNF. The main goal was to assess differences between patients with and without FoF at admission to a SNF, and to assess whether FoF persists after discharge. In addition, the relation between FoF and IADL after discharge was investigated.

## Methods

### Setting and study population

The population studied were older patients who were all newly admitted to rehabilitate in a SNF. Soon after admission to a Dutch SNF, a multidisciplinary rehabilitation plan is made by the elderly care physician; this physician is specially trained in medical care of frail older people and is part of the staff of a nursing home [16]. Patients generally follow a 4–16 weeks rehabilitation programme, which includes treatment of pain and comorbidity, training in ADL, and physical and occupational therapy. Physical therapy involves balance and gait exercises, muscle strengthening and aerobic training. Also walking outdoors and climbing stairs are mostly part of the training. The occupational therapist coaches the patient in daily activities such as getting dressed and going to the toilet. He also assesses whether adaptations at home are required to ensure a safe environment when the patient is discharged. When required, a social worker, psychologist, or a dietician is consulted. Patients are discharged when they can function independently, or with assistance of formal/informal care, at home. Many patients continue some form of physical therapy after discharge.

The present longitudinal observational follow-up study was conducted within the framework of the Back Home study [17]. The Back Home study investigated whether the use of a structured scoring of supporting nursing tasks achieved earlier discharge home for geriatric rehabilitation patients. The study was carried out between October 2011 and November 2012 in four SNFs of the University Network for the Care sector South-Holland. During this period, all newly admitted persons to the SNF were asked to participate in the study. Patients were excluded when they were incompetent to express their will, or were expected to die soon; the elderly care physician assessed whether or not an individual was incompetent.

The Medical Ethics Committee of the Leiden University Medical Center approved the study. Verbal informed consent was obtained from all participants.

### Data collection

Data on FoF were collected at different points in time. These data could be used to assess the prevalence of FoF during admission in the SNF and after discharge, and to analyze the differences between patients with different levels of FoF and no FoF at all.

Within 1 week after admission data were collected on age, gender, living situation, diagnosis, and fall frequency (estimated average number of falls per week). Also, questionnaires and tests were completed, i.e. the Minimal Mental State Examination (MMSE), the Barthel Index, the Self-Efficacy Scale (SES), the one-item instrument for FoF, and the Geriatric Depressions Scale-8 items (GDS8).

At discharge the destination was rated. Participants who were discharged within 17 weeks received a questionnaire 4 weeks after discharge from the SNF. This questionnaire included the one-item FoF scale, the Short Fall-Efficacy Scale-International (FES-I) and the Frenchay Activities Index (FAI).

### Measurement instruments

#### Fear of falling

A one-item FoF instrument was used for follow-up of FoF. The validity of this instrument still requires further research but the reliability of this instrument is good and the instrument has been used in many earlier studies to estimate the prevalence of FoF [14]. It asks one question: “Are you afraid of falling?” and has four answer options: “Not at all”, “A little”, “Quite a bit” and “Very much” [14].

To study FoF after discharge we also used a Fall-Efficacy Scale, i.e. the Short FES-I [18]. The Short FES-I was developed from the FES-I for screening and research purposes. The psychometric properties and discriminative power of the Short FES-I are almost as good as the FES-I [18]. The score on the Short FES-I ranges from 7 to 28, with higher scores indicating more FoF.

#### Cognition

The MMSE is a short screening test for cognitive disorders and dementia [19]. It is widely used in clinical and research settings and has excellent measurement properties [20]. The score ranges from 0 to 30 with higher scores indicating better cognition.

#### Depression

The GDS8 measures depressive symptoms and was developed to screen depression in nursing homes; it is an adaptation of the GDS30 [21]. The score ranges from 0 to 8 with higher scores indicating more depression. The instrument has good measurement properties [21].

#### Activities of daily living (ADL)

ADL were measured with the Barthel Index. The Barthel Index measures independence of a person in doing activities of daily life. Scores of the Barthel Index range from 0 to 20, with higher scores indicating more independence in ADL such as eating, dressing, and going to the toilet [22]. The Barthel Index is widely used and has good measurement properties [23, 24].

#### Self-efficacy

Self-efficacy was measured with the SES [25]. The scale has ten items and higher scores (range 0–30) indicate a higher level of competence to cope with various challenges, such as the confidence to deal with unforeseen circumstances and to find solutions for difficult problems.

#### Instrumental activities of daily living

The FAI was used to assess IADL [26, 27]. It provides a score for the number of times that a person has carried out certain activities (e.g. domestic chores, leisure/work, outdoor activities) and corresponds to the activity/participation domain of the World Health Organization (WHO) International Classification of Function, Disability and Health (ICF) [28]. The FAI consists of 15 questions and every item has a score of 1–4, resulting in a summed score ranging from 15 to 60 [27, 29]. A higher score indicates that the person is more capable in carrying out IADL.

### Statistical analysis

For the analysis patients were divided into two groups based on their answer to the 1-item FoF measure at admission: i) those with no FoF at all, and ii) those with a little, quite a bit and very much FoF. Descriptive measurements such as medians and interquartile ranges (IQR) were used to describe the groups. For continuous data the normality of the distribution was assessed. For normal distributed continuous variables the Student’s *t*-test was used, for non-normal distributed continuous variables the Mann–Whitney *U* test was used. For dichotomous or ordinal variables the Pearson’s Chi-square test was used for independent samples and the McNemar test for correlated samples. A *p*-value <0.05 was used as the cut-off for statistical significance.

Participants who were discharged within 17 weeks after admission and completed the questionnaire sent to them 4 weeks after discharge from the SNF were analysed to assess FoF at admission and after discharge. The McNemar test was used to assess significance. The *T*-test was used for these participants to compare the FAI of participants with and those with no FoF.

Analyses were performed with SPSS for Windows (Version 21, SPSS, Inc., Chicago, IL, USA).

## Results

**Fig. 1:**
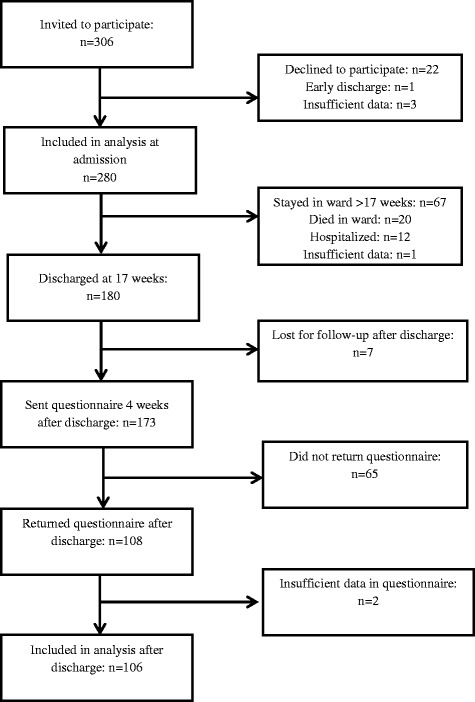
Flow-chart of recruitment and follow-up of participants

Figure 1 presents a flow chart of the participant recruitment and follow-up. Of the 306 patients invited to participate in the study, 22 declined. Of the remaining 284 patients, one participant was discharged almost directly after admission. Subsequently, of the 283 patients who participated, three did not provide sufficient data on FoF and were excluded from the analysis. The majority of participants were women (70.7 %), the median age was 82.4 (IQR: 75.8–87.4) years, and most (70.0 %) lived alone at home before admission to the hospital and the SNF. The underlying diagnosis at admission was: stroke (22.9 %), elective orthopaedic operation (12.9 %), trauma (33.9 %), or another disease (30.4 %). Of all patients, 175 (62.5 %) had a little, quite a bit, or very much FoF at admission.

Table 1 presents the differences between the participants without and with FoF at admission. In the group with FoF, both the median age and the percentage of females were significantly higher. Also, the percentage of participants with stroke was significantly lower (Pearson’s Chi-square test: *p* = 0.040) and with elective orthopaedic surgery was significantly higher in those with FoF (Pearson’s Chi-square test: *p* = 0.043). The GDS8 was significantly higher in the group with FoF (Mann–Whitney *U* test: *p* = 0.029), whereas the SES was significantly higher in the group without FoF (Mann–Whitney *U* test: *p* = 0.043).

**Table 1:** Characteristics of participants without and with fear of falling (FoF) at baseline

	All participants *n* = 280	Participants without FoF *n* = 105	Participants with FoF *n* = 175	*p*-value
Age in years, median (IQR)	82.4 (75.8 – 87.4)	79.7 (72.6 – 85.7)	83.4 (76.9 – 88.1)	0.005*
Female, n (%)	198 (70.7 %)	63 (60.0 %)	135 (77.1 %)	0.002**
Living alone	196 (70.0 %)	69 (65.7 %)	127 (72.6 %)	0.225**
Diagnosis at admission, n (%)				0.017**
- Stroke	64 (22.9 %)	31 (29.5 %)	33 (18.9 %)	0.040***
- Orthopaedic, elective	36 (12.9 %)	8 (7.6 %)	28 (16.0 %)	0.043***
- Trauma	95 (33.9 %)	29 (27.6 %)	66 (37.7 %)	0.084***
- Other	85 (30.4 %)	37 (35.2 %)	48 (27.4 %)	0.169***
Average number of falls per week, median (IQR)	1 (0–3)	0 (0 – 2)	1 (0 – 3)	<0.001*
MMSE, median (IQR)	25 (21–27)	25 (20 – 27)	25 (22 – 27)	0.289*
GDS8 (total), median (IQR)	0 (0–2)	0 (0 – 1)	1 (0 – 2)	0.029*
Barthel at admission, median (IQR)	10 (6–14)	9.5 (6 – 15)	10 (6 – 14)	0.694*
SES (total), median (IQR)	35 (31–38)	35 (33 – 38)	34 (30 – 37)	0.043*

*Mann–Whitney *U* test; **Pearson’s Chi-square test, ***Pearson’s Chi-square test per patient group; *IQR* interquartile range, *MMSE* minimal mental state, *GDS8* Geriatric Depression Scale-8 Items, *SES* self-efficacy scale

At admission to a SNF, FoF was highest in the group with an elective orthopaedic procedure (77.8 %), compared to 69.5 % in those with trauma, 56.5 % in those with other diseases, and 51.6 % in those with stroke (Pearson‘s Chi-square: *p* = 0.017).

At 17 weeks after admission, 67 (23.9 %) of the participants were still in the SNF, 12 (4.3 %) were hospitalised, 20 (7.1 %) had died, and 180 (64.3 %) were discharged. For one patient no data were available at 17 weeks. Of the 180 participants who were discharged, seven were lost to follow-up (five for unknown reasons, while two had died). Of the remaining 173 participants, 108 (62.4 %) returned the questionnaire sent to them 4 weeks after discharge from the SNF (Fig. 1). Of these 108 participants, 95 (88.0 %) were discharged home and 13 (12.0 %) were discharged to a long-term care facility or rehabilitation centre.

Two participants provided no data on FoF after discharge. Of the 106 remaining participants after discharge, 19 (17.9 %) had no FoF, 32 (30.2 %) had little FoF, 28 (26.4 %) had quite a bit, and 27 (25.5 %) had very much FoF. Table 2 shows the changes between FoF at admission and after discharge. At admission, 61 (57.5 %) of these participants had some kind of FoF, whereas after discharge 87 (82.1 %) had FoF (McNemar test: *p* < 0.001).

**Table 2:** Comparison between fear of falling (FoF) at admission to a skilled nursing facility and after discharge home (*n* = 106)

	No FoF after discharge	FoF after discharge	
No FoF at admission	12 (11.3 %)	33 (31.1 %)	45 (42.5 %)
FoF at admission	7 (6.6 %)	54 (50.9 %)	61 (57.5 %)
	19 (17.9 %)	87 (82.1 %)	106 (100.0 %)

When assessing FoF in these 106 participants based on the main patient groups, 78.3, 77.8, 85.4 and 83.3 % of the participants with a stroke (*n* = 23), an elective orthopaedic operation (*n* = 18), a trauma (*n* = 41) or another disease (*n* = 24), respectively, had some kind of FoF 4 weeks after discharge, whereas at admission, 47.8, 66.7, 63.4 and 50.0 % of these participants, respectively, had FoF. These differences were significant for patients with a trauma (McNemar test: *p* = 0.022) and another disease (McNemar test: *p* = 0.008), not for patients with a stroke (McNemar test: *p* = 0.092) and with an elective orthopedic operation (McNemar test: *p* = 0.688).

Table 3 shows the relation between FoF and the FAI, using the score of the total FAI and the scores of the three subscales, i.e. domestic, leisure/work and outdoors [28]. The domestic domain consisted of the first five items of the FAI, the leisure/work domain of items 7, 9, 11 and 13, and the outdoors domain of items 6, 8, 10 and 12. The items 14 and 15 were not included because they do not fit well into any of the three domains [29].

**Table 3:** Instrumental activities of daily living of participants without and with fear of falling (FoF) 4 weeks after discharge

	All participants	Participants without FoF	Participants with FoF	
FAI Total, mean (SD)	28.67 (9.07)	34.84 (8.51)	27.27 (8.70)	T –test for equality of means: *p* = 0.001
- FAI Domestic (SD)	6.49 (5.08)	9.95 (4.09)	5.72 (5.01)	*T*-test for equality of means: *p* < 0.001
- FAI Leisure (SD)	3.21 (2.52)	3.95 (2.37)	3.03 (2.56)	*T*-test for equality of means: *p* = 0.145
- FAI Outdoors (SD)	2.81 (2.81)	4.53 (2.59)	2.45 (2.67)	*T*-test for equality of means: *p* = 0.004

*FAI* Frenchay Activity Index, *SD* standard deviation

A significant relation exists between FoF and the FAI. When assessing the subscales, FoF was significantly related to the domestic domain and to the outdoors domain. The short FES-I of participants with and without FoF after discharge also showed a significant difference, i.e. 17.11 (standard deviation (SD) 5.49) for participants with FoF and 8.65 (SD 2.21) for those without FoF (*T*-test: *p* < 0.001). The Pearson correlation between the short FES-I and the one-item FoF instrument was 0.765 (*p* < 0.001).

## Discussion

FoF is common in older patients who rehabilitate in a SNF of a nursing home. In the present study 62.5 % had FoF at admission. Participants with FoF were more often female and older. Also, they were more often depressed and had a significantly lower self-efficacy. For patients who could be followed-up after discharge, the prevalence of FoF was even higher after discharge. When dividing these patients in different diagnosis groups the increase in FoF after discharge was significant for patients with a trauma and with another disease. Furthermore, the study demonstrated that FoF after discharge was significantly related with IADL.

Although 62.5 % is a relatively high proportion for FoF, it is comparable to another Dutch study investigating patients who rehabilitated in SNF after a hip fracture. In the latter study, 63.0 % had some kind of FoF [30]. In other studies among patients with hip fractures, 50 % indicated to be afraid of falling [31], and 65 % sometimes or often had FoF [32]. In addition, female sex, older age and depression are known risk factors for FoF [33, 34]. These latter factors are also correlated with FoF in long-term care [1].

The present study found that, 4 weeks after discharge from the SNF, the percentage of patients with at least some FoF ranged from 77.8 to 85.4 % for all four groups. This may indicate that, in older persons rehabilitating in a SNF, FoF is more strongly associated with characteristics other than the underlying health condition itself. More studies are needed to establish whether this is related to the vulnerable condition and the high number of comorbidities in these older patients, or due to the ageing process itself [35, 36].

FoF has rarely been assessed longitudinally. Therefore, our remarkable finding that the prevalence of FoF increases four weeks after discharge needs to be further evaluated over longer periods of time. A study in community-dwelling older adults, in which the 24-month cumulative incidence of FoF was 45.4 %, found that FoF can persist over time [37]. Predictors for persistent FoF in this latter study were depressive symptoms, clinical gait abnormality, female sex and previous falls; all these factors are reported to be related to vulnerability [38]. Depression, female sex, and average number of falls were also characteristics in our study which were related to FoF.

A possible explanation for the increase of FoF after discharge is that patients cannot immediately oversee all possible consequences, but are confronted with their shortcomings at home. Also, when patients are rehabilitating in a SNF, they encounter substantial physical, psychological and social support during admission. Particularly because 70 % of these patients lived alone, this support will have been missed after discharge, which may have enhanced FoF.

While FoF has been identified as an obstacle for rehabilitation after hip fracture [6, 7], more recently FoF has also been regarded as an emerging issue in other diseases, such as a stroke [39, 40]. For example, Schmid et al. , assessed FoF directly after stroke and 6 months later. In that study (which also used a one-item instrument), FoF at baseline was 54 % [41]; after 6 months, 7 (39 %) of the 18 patients that could be followed-up had some FoF. Unfortunately, that study included only 18 patients with a 6-month follow-up and the characteristics of the group were different from those of our participants. Only participants from a single, university-based, teaching hospital were recruited, with a mean age of 59 years, and 64 % of the participants were male [41]. In another study from Korea, in which FoF was assessed in sub-acute stroke patients (3–6 months of stroke duration), 18 of the 34 (53 %) patients reported to have FoF [39]. The results of these studies are in line with the prevalence of FoF among stroke patients in our study, in which about half of the patients with a stroke, i.e. 33 of the 64 patients (51.6 %), reported FoF at admission. In a qualitative study three factors were possibly associated with the development of post-stroke FoF: a) an initial fall coinciding with the stroke onset, b) perception of post-stroke body changes, and c) a pervasive everyday fear of future falls [40]. Particularly the post-stroke body changes may explain the rather high and persistent prevalence of FoF in stroke patients, even after discharge home.

FoF is particularly important because, as shown in the present study, it is directly related to conducting more complex activities. FoF may hamper IADL after discharge. Feared consequences of falling such as loss of functional independence and damage to identity (i.e. through social embarrassment and indignity) are reported to be correlated with avoidance of activity [2]. When dividing FoF into three components, i.e. physiological, behavioural and cognitive, particularly the behavioural component of FoF of self-restricted avoidance of activities, may lead to a negative spiral toward frailty and increased dependency in these discharged patients [12].

A study by Denkinger et al. [5] demonstrated that falls-related self-efficacy is the only parameter that significantly predicts rehabilitation outcome at discharge and follow-up across outcomes such as ADL, gait and function. In our study we also demonstrated that falls-related self-efficacy is related with IADL after discharge, particularly with the domestic and outdoors domain of the FAI. Hence, prevention and treatment of FoF is an important clinical issue and therapists should be aware of the relation between FoF and the effects on recovery [40]. In addition, it is important to develop and study specific interventions which target falls-related self-efficacy, as a modifiable factor during rehabilitation, impacting on FoF and IADL after discharge. Since FoF can be rather persistent, such programmes need to be continued after discharge from the SNF.

The 1-item FoF instrument, which has been used in many earlier studies as a simple and reliable instrument to measure FoF, has some flaws [14]. When used dichotomous to distinguish between participants with no FoF and some kind of FoF, it does not allow for any variability in degrees of FoF. The 1-item instrument also does not differentiate between different types of activities for which FoF may be present. It is often used as an umbrella instrument for FoF, not distinguishing between the different aspects of FoF, e.g. physiological, behavioral and cognitive elements [12]. Nevertheless this instrument has the advantage of being straightforward and its ease of generating prevalence estimates [8].

A strength of our study is that FoF was measured at two different points in time, not only during admission but also after discharge. Also, FoF was measured by different instruments with good measurement properties. We found a strong relation between the different instruments for FoF; the Pearson’s correlation was 0.765. The fact that these instruments may measure somewhat different constructs has been extensively discussed [14]. The short FES-I, which measures ‘concern’ about falling may focus more on the cognitive elements of FoF and less on emotional aspects [12, 13]. IADL were also assessed with a validated and commonly used instrument, i.e. the Frenchay Activity Index.

Another strength of our study is that the included patients had different types of underlying conditions (e.g. trauma and stroke) and that we focused on vulnerable older patients who may be more susceptible for FoF. These patients are often excluded from studies on rehabilitation [6]. Furthermore, the 60 % response to the questionnaires by the discharged participants is relatively high.

A weakness of the study is that not all patients could be followed-up. No further data were collected for patients who were still not discharged from a SNF after 17 weeks.

## Conclusion and future directions

FoF is highly prevalent and increased in older patients rehabilitating in a SNF. At 4 weeks after discharge, FoF was associated with IADL. Therefore, interventions are needed to reduce FoF and enhance IADL after discharge. Such interventions should be further developed and studied in older vulnerable persons who rehabilitate in SNFs.

## Abbreviations

ADL: activities of daily living
FAI: Frenchay Activity Index
FES-I: Falls Efficacy Scale – International
FoF: fear of falling
GDS8: Geriatric Depression Scale-8 Items
IADL: instrumental activities of daily living
ICF: International Classification of Function, Disability and Health
IQR: interquartile range
MMSE: Minimal Mental State Examination
SD: standard deviation
SES: self-efficacy scale
SNF: skilled nursing facility
WHO: World Health Organization

## References

[1] Lach HW, Parsons JL (2013). Impact of fear of falling in long term care: an integrative review. J Am Med Dir Assoc..

[2] Yardley L, Smith H (2002). A prospective study of the relationship between feared consequences of falling and avoidance of activity in community-living older people. The Gerontologist.

[3] Scheffer AC, Schuurmans MJ, van Dijk N, van der Hooft T, de Rooij SE (2008). Fear of falling: measurement strategy, prevalence, risk factors and consequences among older persons. Age Ageing.

[4] Lach HW (2005). Incidence and risk factors for developing fear of falling in older adults. Public Health Nurs.

[5] Denkinger MD, Igl W, Lukas A, Bader A, Bailer S, Franke S, Denkinger CM, Nikolaus T, Jamour M (2010). Relationship between fear of falling and outcomes of an inpatient geriatric rehabilitation population – fear of the fear of falling. J Am Geriatr Soc.

[6] Visschedijk J, Achterberg W, van Balen R, Hertogh C (2010). Fear of falling after hip fracture: a systematic review of measurement instruments, prevalence, interventions, and related factors. J Am Geriatr Soc.

[7] Oude Voshaar RC, Banerjee S, Horan M, Balwin R, Pendleton N, Proctor R, Tarrier N, Woodward Y, Brums A (2006). Fear of falling more important than pain and depression for functional recovery after surgery for hip fracture in older people. Psychol Med.

[8] Legter K (2002). Fear of falling. Phys Ther.

[9] Tinetti ME, Powell L (1993). Fear of falling and low self-efficacy: a case of dependence in elderly persons. J Gerontol.

[10] Maki BE, Holliday PJ, Topper AK (1991). Fear of falling and postural performance in the elderly. J Gerontol A Biol Sci Med Sci.

[11] Mckee KJ, Orbell S, Austin CA, Bettridge R, Liddle BJ, Morgan K, Radley K (2002). Fear of falling, falls efficacy, and health outcomes in older people following a hip fracture. Disabil Rehabil.

[12] Hadjistavropoulos T, Delbaer K, Fitzgerald TD (2011). Reconceptualizing the role of fear of falling and balance confidence in fall risk. J Aging Health.

[13] Yardley L, Beyer N, Hauer K, Kempen G, Piot-Ziegler C, Todd C (2005). Development and initial validation of the Falls Efficacy Scale-International (FES-I). Age Ageing.

[14] Jorstad E, Hauer K, Becker C, Lamb SE, Group PFNE (2005). Measuring the psychological outcomes of falling: a systematic review. J Am Geriatr Soc.

[15] Holstege MS, Zekveld IG, Caljouw MA, Peerenboom PB, van Balen R, Gussekloo J, Achterberg WP (2013). Relationship of patient volume and service concentration with outcome in geriatric rehabilitation. J Am Med Dir Assoc.

[16] Koopmans RT, Lavrijsen JC, Hoek JF, Went PB, Schols JM (2010). Dutch elderly care physician: a new generation of nursing home physician specialists. J Am Geriatr Soc.

[17] Caljouw MAA, Bakkers E, Holstege MS, van Balen R, Achterberg WP (2014). Structured scoring of supporting nursing tasks in post-acute care to enhance early supported discharge in geriatric rehabilitation. The BACK-HOME study. Eur Geriatr Med.

[18] Kempen GI, Yardley L, van Haastregt JC, Zijlstra GA, Beyer N, Hauer K, Todd C (2008). The Short FES-I: a shortened version of the falls efficacy scale-international to assess fear of falling. Age Ageing.

[19] Folstein MF, Folstein SE, McHugh PR (1975). “Mini-mental state”. A practical method for grading the cognitive state of patient for the clinician. J Psychiatr Res.

[20] Tombaugh TN, McIntyre NJ (1992). The mini-mental state examination: a comprehensive review. J Am Geriatr Soc.

[21] Jongenelis K, Gerritsen DL, Pot AM, Beekman AT, Eisses AM, Kluiter H, Ribbe MW (2007). Construction and validation of a patient- and user-friendly nursing home version of the Geriatric Depression Scale. Int J Geriatr Pscyhiatr.

[22] Mahony FI, Barthel DW (1965). Functional Evaluation: The Barthel Index. Md Stat Med J.

[23] Roy CW, Togneri J, Hay E, Pentland B (1988). An interrater reliability study of the Barthel Index. Int J Rehabil Res.

[24] Quinn TJ, Langhorne P, Stott DJ (2011). Barthel Index for stroke trials: development, properties, and application. Stroke.

[25] Teeuw B, Schwarzer R, Jerusalem M. Dutch adaptation of the general self-efficacy scale [Internet]. 1994 [cited 2012 Oct 2]. Available from: http://userpage.fu-berlin.de/~health/dutch.htm.

[26] van der Zee CH, Kap A, Rambaran Mishre R, Schouten EJ, Post MW (2011). Responsiveness of four participation measures to changes during and after outpatient rehabilitation. J Rehabil Med.

[27] Schuling J, de Haan R, Limburg M, Groenier KH (1993). The Frenchay Activities Index. Assessment of functional status in stroke patients. Stroke.

[28] World Health Organization (WHO) (2007). International Classification of Functioning, Disability and Health: ICF.

[29] Holbrook M, Skilbeck C (1983). An activities index for use with stroke patients. Age Ageing.

[30] Visschedijk J, van Balen R, Hertogh C, Achterberg W (2013). Fear of falling in patients with hip fractures: Prevalence and related psychological factors. J Am Med Dir Assoc..

[31] Muche R, Eichner GF, Kinzl L, Becker C (2003). Risikofaktoren und prognosemöglichkeiten für mortalität und soziofunktionelle Einschränkungen bei Älteren nach proximalen Femurfrakturen. Euro J Ger.

[32] Ingemarsson AH, Frändin K, Hellström K, Rundgren A (2000). Balance function and fall-related efficacy in patients with a newly operated hip fracture. Clin Rehabil.

[33] Friedman SM, Munoz B, West SK, Rubin GS, Fried LP (2002). Falls and fear of falling: which comes first? A longitudinal prediction model suggests strategies for primary and secondary prevention. J Am Geriatr Soc.

[34] Austin N, Devine A, Dick I, Prince R, Bruce D (2007). Fear of falling in older women: a longitudinal study of incidence, persistence, and predictors. J Am Geriatr Soc.

[35] Spruit-van Eijk M, Zuidema SU, Buijck BI, Koopmans RT, Geurts AC (2012). Determinants of rehabilitation outcome in geriatric patients admitted to skilled nursing facilities after stroke: a Dutch multi-centre cohort study. Age Ageing.

[36] Clegg A, Young J, Illife S, Olde Rikkert M, Rockwood K (2013). Frailty in elderly people. Lancet.

[37] Oh-Park M, Xue X, Holtzer R, Verghese J (2011). Transient versus persistent fear of falling in community-dwelling older adults: incidence and risk factors. J Am Geriatr Soc.

[38] Gobbens RJ, van Assen MA, Luijkx KG, Wijnen-Sponselee MT, Schols JM (2010). The Tilburg Frailty Indicator: psychometric properties. J Am Med Dir Assoc.

[39] Kim EJ, Kim DY, Kim WH, Lee KL, Yoon YH, Park JM, Shin JL, Kim SK, Kim DG (2012). Fear of falling in subacute hemiplegic stroke patients: associating factors and correlations with quality of life. Ann Rehabil Med.

[40] Schmid AA, Rittman M (2007). Fear of falling: an emerging issue after stroke. Top Stroke Rehabil.

[41] Schmid A, van Puymbroeck M, Knies K, Sprangler-Morris WK, Damush T, Williams L (2011). Fear of falling among people who have sustained a stroke: a 6-month longitudinal pilot study. Am J Occup Ther.

